# Sociodemographic and Occupational Factors Associated with Low Early Uptake of COVID-19 Vaccine in Hospital-Based Healthcare Workers, Georgia, March–July 2021

**DOI:** 10.3390/vaccines10081197

**Published:** 2022-07-27

**Authors:** Héloïse Lucaccioni, Giorgi Chakhunashvili, Carl Jason McKnight, Tamila Zardiashvili, Pernille Jorgensen, Richard Pebody, Esther Kissling, Mark A. Katz, Lia Sanodze

**Affiliations:** 1 European Programme for Intervention Epidemiology Training, European Centre for Disease Prevention and Control, 171 83 Stockholm, Sweden; 2 National Center for Disease Control and Public Health, 0198 Tbilisi, Georgia; g.chakhunashvili@ncdc.ge (G.C.); l.sanodze@ncdc.ge (L.S.); 3 South Caucasus Hub, World Health Organization Regional Office for Europe, 0179 Tbilisi, Georgia; mcknightcj@who.int; 4 Country Office, World Health Organization, 0179 Tbilisi, Georgia; zardiashvilit@who.int; 5 World Health Organization Regional Office for Europe, DK-2100 Copenhagen, Denmark; jorgensenp@who.int (P.J.); pebodyr@who.int (R.P.); katzm@who.int (M.A.K.); 6 Epiconcept, 75011 Paris, France; e.kissling@epiconcept.fr

**Keywords:** COVID-19, vaccine, health workers, hesitancy, Republic of Georgia, public health promotion

## Abstract

In Georgia, an upper-middle income European country, the COVID-19 vaccine rollout began on 15 March 2021 with health workers (HWs), a priority group for vaccination. We assessed the factors associated with COVID-19 vaccination among HWs at six large hospitals in the early stages of the vaccine rollout (March–July 2021). Among 1533 HWs, 274 (17.9%) had received one dose of the COVID-19 vaccine. Strong independent predictors of early vaccine uptake were age > 40 years, especially 50–59 years old (aOR 2.40, 95% CI 1.50–3.88), considering the vaccine as “somewhat effective” or “very effective” rather than “not effective” (aOR 6.33, 95% CI 2.29–26.3 and aOR 10.9, 95% CI 3.88–45.70, respectively), and previous vaccination against seasonal influenza (aOR 2.98, 95% CI 2.19–4.08). Previous SARS-CoV-2 infection was negatively associated with receiving the vaccine (aOR 0.6, 95% CI 0.40–0.80). Compared to physicians, nurses/midwives (aOR 0.22, 95% CI 0.15–0.32), administrative staff (aOR 0.36, 95% CI 0.22–0.56), and ancillary staff (aOR 0.07, 95% CI 0.04–0.15) were less likely to have received the COVID-19 vaccine. Tailoring the COVID-19 vaccine communications campaign to younger and non-physician HWs, and emphasizing the benefits of the COVID-19 vaccine, could help further increase vaccine coverage among HWs in Georgia.

## 1. Introduction

COVID-19 vaccine uptake has been variable among health workers (HWs) in Europe; rates of vaccine coverage among HWs have been particularly low in some middle- and low-income countries in the eastern part of the European region [1,2].

While lack of vaccine availability has partly contributed to this low uptake, vaccine hesitancy among HWs continues to be a challenge [3,4,5]. Differences in uptake by occupation, gender, and age have been observed in other geographical settings [6,7,8,9,10,11]. Understanding the reasons for low vaccine uptake among HWs is critical for targeting public health efforts to increase uptake in this important population.

HWs play a critical role in the pandemic response. They are at high risk of infection due to occupational exposure and, furthermore, infected HWs who come to work can transmit the virus to vulnerable patients [12]. In addition, HWs are a primary, professional trusted source of information, and therefore vaccine hesitancy among HWs can lead to hesitancy among their patients [13,14].

In the Republic of Georgia, an upper-middle income country of 3.7 million people, the COVID-19 vaccine rollout began in Georgia on 15 March 2021 [15]. The first vaccines, 43,200 doses of AstraZeneca vaccine and 30,420 doses of Pfizer-BioNTech vaccine, were procured via the COVAX facility mechanism, the global delivery and procurement pillar of the Access to COVID-19 Tools (ACT) Accelerator initiative. Later in 2021, additional vaccines (1,700,000 doses of Sinopharm vaccine and 1,100,000 doses of Sinovac vaccine from April, and 1,000,000 additional doses of Pfizer-BioNTech vaccine from June) were procured by the government of Georgia outside of the COVAX mechanism. The national COVID-19 vaccine plan aimed to vaccinate 60% (1.7 million) of the adult population by the end of 2021, and, like in many other countries, prioritized health workers for the vaccination initially [16]. The plan set a vaccination target of 46,000 HWs (out of 71,000 total HWs) in the first five weeks of the rollout. Vaccination was offered to all eligible HWs regardless of age or occupation. As of 24 January 2022, only 71.3% of HWs had completed a primary COVID-19 vaccine series [17].

In March 2021, the National Center for Disease Control and Public Health of Georgia (NCDC) and the World Health Organization (WHO) initiated a prospective cohort study to measure the effectiveness of the COVID-19 vaccine among hospital-based HWs [18]. We analyzed enrolment data from this cohort study to assess socio-demographic, clinical, occupational, and behavioral factors, and knowledge and attitudes about the COVID-19 vaccine associated with early uptake of the COVID-19 vaccine.

## 2. Materials and Methods

The study was initially a one-year (March 2021–April 2022) prospective cohort study among HWs at six large hospitals in Tbilisi and Batumi, to evaluate the effectiveness of the COVID-19 vaccine in preventing laboratory-confirmed SARS-CoV-2 infection [18]. The study was approved by the NCDC and WHO Ethical Review Committees.

From 19 March–16 July 2021, we invited all HWs (clinical and non-clinical staff) over 18 years old who were employed at the study sites and eligible to receive the COVID-19 vaccine to participate in the study. HWs could enroll voluntarily in the study regardless of whether they had already received a dose of the COVID-19 vaccine and regardless of their intention to get vaccinated. At the time of enrolment, contra-indications to the COVID-19 vaccine in Georgia included having a previous SARS-CoV-2 infection <120 days ago, and acute febrile illness [19]. At the time of enrolment, participants completed a questionnaire that included questions about socio-demographic and clinical information, occupation, prior SARS-CoV-2 infection (self-reported positive Reverse Transcription–Polymerase Chain Reaction (RT-PCR) or Rapid Antigen Test (RAT) test result), recent behavior with respect to public health and social health measures (e.g., use of facemasks, physical distancing, gathering in groups >10 people, use of public transports, receiving or visiting others indoor), knowledge and attitudes about COVID-19 vaccine, and COVID-19 and seasonal influenza vaccination history. Study staff verified participants’ vaccination status through the National Immunization Registry or individual vaccination cards. All data were systematically entered into REDCap, a data management platform [20].

### Data Analysis

The primary outcome for this study was vaccination status (i.e., vaccinated/unvaccinated with the first dose of a COVID-19 vaccine) on the day of enrolment, irrespective of vaccine brand. Independent variables were self-reported socio-demographic characteristics, clinical and behavioral factors, and knowledge and attitudes about the COVID-19 vaccination.

We excluded participants with unknown vaccination status and participants who were not eligible for vaccination before their enrolment in the study due to a SARS-CoV-2 infection in the previous 120 days.

We computed descriptive statistics as frequency and percentage for categorical variables, or as median and inter-quartile range for continuous variables. We conducted a univariable analysis using a chi-squared test for independence or Wilcoxon rank sum test, as appropriate, followed by logistic regression modeling to measure crude associations between each independent variable and the outcome of vaccination status at enrolment.

We then conducted a backward multivariable analysis to assess factors associated with early COVID-19 vaccine uptake, adjusting for socio-demographic, clinical, and behavioral factors, and knowledge and attitudes about the COVID-19 vaccine. The hospital study site was included a priori. We included in the initial model all independent variables with a *p*-value < 0.2 in the univariable analysis, and sequentially removed the least significant variable until only significant variables remained. We selected the best fit model by examining the Bayesian Information Criterion (BIC) score of successive nested models and retaining the model with the lowest BIC score. We calculated crude and adjusted odds ratios (aOR) and their 95% confidence intervals (95% CI). We conducted a collinearity diagnostic using the variance inflation factor (VIF).

For the descriptive and univariable analysis, we included all available observations. However, we performed a complete case analysis in the multivariable models, excluding 4 (0.3%) observations with missing data.

To further investigate factors associated with vaccine uptake among non-physicians, we conducted a similar but separate analysis with subjects restricted to nurses and midwives.

Analyses were conducted in R statistical software (v.4.1.2; R Core Team, Vienna, Austria) [21].

## 3. Results

Of the 1606 participants enrolled in the study, we included 1533 participants in this analysis. We excluded 73 (4.5%) participants because of unknown vaccination status (n = 16), or ineligibility to receive the COVID-19 vaccine due to a previous SARS-CoV-2 infection <120 days before enrolment (n = 55) or at an unknown date (n = 2).

Characteristics of participants, both overall and stratified by vaccination status, with crude and adjusted measures of associations are presented in Table 1.

Most participants were female (1289; 84.1%), and the median age was 41 years old (interquartile range: 29–53 years); 617 (40.2%) were nurses or midwives, 314 (20.5%) were physicians, 241 (15.7%) were ancillary workers, and 227 (14.8%) were administrative staff. Over half of the participants provided direct medical care to patients (800; 52.2%). Most participants reported being “well informed” (970; 63.3%) or “extremely well informed” (282; 18.4%) about the COVID-19 vaccination, but less than a third (465; 30.3%) said that the COVID-19 vaccination is “very effective” at preventing COVID-19 disease, and only half (798; 52.1%) “mostly agreed” or “totally agreed” that COVID-19 vaccination is safe.

Overall, 274 (17.9%) participants had received one vaccine dose at enrolment; 172 (62.8%) received Pfizer-BioNTech vaccine, 50 (18.2%) received Sinovac, 33 (12.0%) received Sinopharm, and 19 (6.9%) received AstraZeneca.

After controlling for hospital study site in the multivariable analysis, occupation, seasonal influenza vaccination, age groups, and opinion about the effectiveness of COVID-19 vaccination remained significantly associated with vaccination status. However, sex, underlying medical condition, regular contact with pregnant women or infants at work, providing direct care to a patient, performing respiratory procedures, and other behaviors and knowledge and attitudes about the COVID-19 vaccine were not retained in the final model. Non-physicians were less likely to be vaccinated compared to physicians, particularly nurses (aOR 0.22, 95% CI 0.15–0.32), administrative staff (aOR 0.36, 95% CI 0.22–0.56), and ancillary staff (aOR 0.07, 95% CI 0.04–0.15). Participants who had a previous SARS-CoV-2 infection were less likely to have received a vaccine compared to those who had not been infected (aOR 0.57, 95% CI 0.42–0.78). In contrast, having received the seasonal influenza vaccine during the 2020–2021 influenza season (aOR 2.98, 95% CI 2.19–4.08), judging the COVID-19 vaccine as “somewhat effective” or “very effective” (aOR 6.33, 95% CI 2.29–26.3; and aOR 10.9, 95% CI 3.88–45.7, respectively), and being in an age group > 40 years old, especially 50–59 years old (aOR 2.40, 95% CI 1.50–3.88), were strong independent predictors of COVID-19 vaccine uptake.

Results from the final model restricted to nurses and midwives remained consistent with findings from the overall cohort (Table 2). Nurses and midwives who considered the COVID-19 vaccine as “highly effective” were more likely to be vaccinated (aOR 6.78, 95% CI 1.84–44.20) compared to those who considered the vaccine “not effective”, and those previously vaccinated against seasonal influenza were twice as likely to have received the COVID-19 vaccine (aOR 2.38, 95% CI 1.40–4.07). Nurses and midwives who had regular contact with infants at work were also more likely to have received the COVID-19 vaccine (aOR 3.56, 95% CI 1.37–9.01). Previous SARS-CoV-2 infection and age groups were not significantly associated with vaccine uptake among nurses and midwives and were not retained in this final model.

In both models (all HWs and nurses/midwives only), the variance inflation factor showed low correlations among the investigated variables (VIF < 2).

**Table 1:** Characteristics of health workers and factors associated with early COVID-19 uptake in the univariable and multivariable analysis, Georgia, March–July 2021.

		Vaccination Status				Crude Odds Ratio (OR) N = 1533		Adjusted Odds Ratio (aOR) N = 1529	
Characteristic	Missing n (%)	Total Study Population (%) N = 1533	Number Unvaccinated (%) N = 1259	Number Vaccinated (%) N = 274	*p*-Value ^2^	OR (95% CI) ^3^	*p*-Value	aOR (95% CI) ^3^	*p*-Value
**Basic Characteristics**									
Hospital study site	0 (0%)				**<0.001**				
Acad. K Central University Hosp.		280 (18.3%)	227 (18.0%)	53 (19.3%)		-		-	
Batumi Republican Hospital		305 (19.9%)	224 (17.8%)	81 (29.6%)		1.55 (1.05, 2.30)	**0.029**	1.17 (0.74, 1.86)	0.5
Bochorishvili Clinic		183 (11.9%)	164 (13.0%)	19 (6.9%)		0.50 (0.28, 0.86)	**0.014**	0.60 (0.32, 1.11)	0.11
Bokeria Tbilisi Referral Hospital		302 (19.7%)	260 (20.7%)	42 (15.3%)		0.69 (0.44, 1.07)	0.10	0.57 (0.34, 0.95)	0.033
Caucasus Medical Centre		289 (18.9%)	244.0 (19.4%)	45 (16.4%)		0.79 (0.51, 1.22)	0.3	0.81 (0.49, 1.33)	0.4
Infectious Disease Hospital		174 (11.4%)	140 (11.1%)	34 (12.4%)		1.04 (.64, 1.67)	0.9	0.64 (0.36, 1.12)	0.12
Age group (years)	0 (0%)				**<0.001**				
18–29		386 (25.2%)	343 (27.2%)	43 (15.7%)		-		-	
30–39		334 (21.8%)	282 (22.4%)	52 (19.0%)		1.47 (0.95, 2.28)	0.081	1.22 (0.76, 1.97)	0.4
40–49		321 (20.9%)	258 (20.5%)	63 (23.0%)		1.95 (1.28, 2.98)	**0.002**	1.83 (1.14, 2.94)	**0.013**
50–59		312 (20.4%)	241 (19.1%)	71 (25.9%)		2.35 (1.56, 3.57)	**<0.001**	2.40 (1.50, 3.88)	**<0.001**
60+		180 (11.7%)	135 (10.7%)	45 (16.4%)		2.66 (1.67, 4.23)	**<0.001**	1.90 (1.10, 3.27)	**0.021**
Sex	0 (0%)				0.5				
Female		1289 (84.1%)	1062 (84.4%)	227 (82.8%)		-			
Male		244 (15.9%)	197 (15.6%)	47 (17.2%)		1.12 (0.78, 1.57)	0.5		
Occupation *	0 (0%)				**<0.001**				
Physicians		314 (20.5%)	184 (14.6%)	130 (47.4%)		-		-	
Nurses and midwives		617 (40.2%)	540 (42.9%)	77 (28.1%)		0.20 (0.14, 0.28)	**<0.001**	0.22 (0.15, 0.32)	**<0.001**
Ancillary workers		241 (15.7%)	225 (17.9%)	16 (5.8%)		0.10 (0.06, 0.17)	**<0.001**	0.08 (0.04, 0.15)	**<0.001**
other health professionals		88 (5.7%)	78 (6.2%)	10 (3.6%)		0.18 (0.09, 0.35)	**<0.001**	0.15 (0.07, 0.30)	**<0.001**
Administrative workers		227 (14.8%)	188 (14.9%)	39 (14.2%)		0.29 (0.19, 0.44)	**<0.001**	0.36 (0.22, 0.56)	**<0.001**
Unspecified/unknown occupation		46 (3.0%)	44 (3.5%)	2 (0.7%)		0.06 (0.01, 0.21)	**<0.001**	0.07 (0.01, 0.27)	**<0.001**
Household size ^1^	0 (0%)	4.0 (3.0, 5.0)	4.0 (3.0, 5.0)	4.0 (3.0, 5.0)	>0.9	1.00 (0.91, 1.09)	>0.9		
Underlying condition **	0 (0%)				**0.038**				
None		1155 (75.3%)	962 (76.4%)	193 (70.4%)		-			
One or more		378 (24.7%)	297 (23.6%)	81 (29.6%)		1.36 (1.01, 1.81)	**0.038**		
Smoking status	2 (0.1%)				0.7				
Never smoked		1018 (66.5%)	839 (66.7%)	179 (65.3%)		-			
Current/previous smoker		513 (33.5%)	418 (33.3%)	95 (34.7%)		1.07 (0.81, 1.40)	0.7		
Self-rated health status	0 (0%)				0.6				
Poor		19 (1.2%)	18 (1.4%)	1 (0.4%)		-			
Average/normal		634 (41.4%)	520 (41.3%)	114 (41.6%)		3.95 (0.80, 71.4)	0.2		
Good (better than average)		519 (33.9%)	424 (33.7%)	95 (34.7%)		4.03 (0.82, 73.0)	0.2		
Excellent		361 (23.5%)	297 (23.6%)	64 (23.4%)		3.88 (0.78, 70.4)	0.2		
Vaccinated against influenza	1 (<0.1%)				**<0.001**				
No		1046 (68.3%)	920 (73.1%)	126 (46.0%)		-		-	
Yes		486 (31.7%)	338 (26.9%)	148 (54.0%)		3.20 (2.45, 4.19)	**<0.001**	2.98 (2.19, 4.08)	**<0.001**
Previous SARS-CoV-2 infection	0 (0%)				**<0.001**				
No		835 (54.5%)	660 (52.4%)	175 (63.9%)		-		-	
Yes		698 (45.5%)	599 (47.6%)	99 (36.1%)		0.62 (0.47, 0.81)	**<0.001**	0.57 (0.42, 0.78)	**<0.001**
Service types									
Regular contact with infants at work	0 (0%)				**0.019**				
No		1389 (90.6%)	1151 (91.4%)	238 (86.9%)		-			
Yes		144 (9.4%)	108 (8.6%)	36 (13.1%)		1.61 (1.07, 2.39)	**0.020**		
Regular contact with elderly (>65 years) at work	0 (0%)				>0.9				
No		700 (45.7%)	575 (45.7%)	125 (45.6%)		-			
Yes		833 (54.3%)	684 (54.3%)	149 (54.4%)		1.00 (0.77, 1.30)	>0.9		
Regular contact with pregnant women at work	0 (0%)				0.051				
No		1361 (88.8%)	1127 (89.5%)	234 (85.4%)		-			
Yes		172 (11.2%)	132 (10.5%)	40 (14.6%)		1.46 (0.99, 2.12)	0.052		
Provides direct patient care	0 (0%)				**<0.001**				
No		733 (47.8%)	627 (49.8%)	106 (38.7%)		-			
Yes		800 (52.2%)	632 (50.2%)	168 (61.3%)		1.57 (1.21, 2.06)	**<0.001**		
Performs respiratory procedures ***	0 (0%)				0.071				
No		919 (59.9%)	768 (61.0%)	151 (55.1%)		-			
Yes		614 (40.1%)	491 (39.0%)	123 (44.9%)		1.27 (0.98, 1.66)	0.072		
Attitude towards the vaccination									
How informed are you about COVID-19 vaccine?	0 (0%)				**<0.001**				
Slightly informed		84 (5.5%)	80 (6.4%)	4 (1.5%)		-			
Somewhat informed		197 (12.9%)	181 (14.4%)	16 (5.8%)		1.77 (0.63, 6.32)	0.3		
Well informed		970 (63.3%)	790 (62.7%)	180 (65.7%)		4.56 (1.87, 15.1)	**0.003**		
Extremely well informed		282 (18.4%)	208 (16.5%)	74 (27.0%)		7.12 (2.83, 23.9)	**<0.001**		
COVID-19 vaccination is safe	0 (0%)				**<0.001**				
Mostly disagree		181 (11.8%)	151 (12.0%)	30 (10.9%)		-			
Neutral		554 (36.1%)	508 (40.3%)	46 (16.8%)		0.46 (0.28, 0.75)	**0.002**		
Mostly agree		238 (15.5%)	210 (16.7%)	28 (10.2%)		0.67 (0.38, 1.17)	0.2		
Totally agree		560 (36.5%)	390 (31.0%)	170 (62.0%)		2.19 (1.44, 3.43)	**<0.001**		
COVID-19 vaccination is effective	0 (0%)				**<0.001**				
Not effective		185 (12.1%)	182 (14.5%)	3 (1.1%)		-		-	
Somewhat effective		883 (57.6%)	746 (59.3%)	137 (50.0%)		11.1 (4.16, 45.50)	**<0.001**	6.33 (2.29, 26.30)	**0.002**
Very effective		465 (30.3%)	331 (26.3%)	134 (48.9%)		24.6 (9.13, 101)	**<0.001**	10.9 (3.88, 45.70)	**<0.001**
Infection prevention behaviors									
Wears a mask indoors	1 (<0.1%)				0.3				
Never or rarely		20 (1.3%)	18 (1.4%)	2 (0.7%)		-			
Episodically		22 (1.4%)	21 (1.7%)	1 (0.4%)		0.43 (0.02, 4.83)	0.5		
Often		182 (11.9%)	153 (12.2%)	29 (10.6%)		1.71 (0.46, 11.1)	0.5		
Always		1308 (85.4%)	1066 (84.7%)	242 (88.3%)		2.04 (0.58, 12.9)	0.3		
Maintains physical distance (>2 m) with others indoor	3 (0.2%)				**0.040**				
Never or rarely		90 (5.9%)	81 (6.4%)	9 (3.3%)		-			
Episodically		234 (15.3%)	202 (16.1%)	32 (11.7%)		1.43 (0.68, 3.30)	0.4		
Often		507 (33.1%)	410 (32.6%)	97 (35.4%)		2.13 (1.09, 4.69)	**0.041**		
Always		699 (45.7%)	563 (44.8%)	136 (49.6%)		2.17 (1.12, 4.75)	**0.033**		
Uses public transport	0 (0%)				**<0.001**				
Never		388 (25.3%)	288 (22.9%)	100 (36.5%)		-			
1 to 2 times/week		338 (22.0%)	278 (22.1%)	60 (21.9%)		0.62 (0.43, 0.89)	**0.010**		
3 to 5 times/week		371 (24.2%)	307 (24.4%)	64 (23.4%)		0.60 (0.42, 0.85)	**0.005**		
>5 times/week		436 (28.4%)	386 (30.7%)	50 (18.2%)		0.37 (0.26, 0.54)	**<0.001**		
Gathers in groups (>10 people)	0 (0%)				0.11				
Never		854 (55.7%)	698 (55.4%)	156 (56.9%)		-			
1 to 2 times/week		490 (32.0%)	394 (31.3%)	96 (35.0%)		1.09 (0.82, 1.44)	0.5		
3 to 5 times/week		112 (7.3%)	99 (7.9%)	13 (4.7%)		0.59 (0.31, 1.04)	0.084		
>5 times/week		77 (5.0%)	68 (5.4%)	9 (3.3%)		0.59 (0.27, 1.15)	0.2		
Receives visitors indoors	0 (0%)				**0.004**				
Never		139 (9.1%)	113 (9.0%)	26 (9.5%)		-			
Rarely		1061 (69.2%)	851 (67.6%)	210 (76.6%)		1.07 (0.69, 1.72)	0.8		
Episodically		231 (15.1%)	201 (16.0%)	30 (10.9%)		0.65 (0.37, 1.16)	0.14		
Often		102 (6.7%)	94 (7.5%)	8 (2.9%)		0.37 (0.15, 0.82)	**0.020**		
Visits others indoors	0 (0%)				**0.011**				
Never		244 (15.9%)	188 (14.9%)	56 (20.4%)		-			
Rarely		1065 (69.5%)	872 (69.3%)	193 (70.4%)		0.74 (0.53, 1.05)	0.084		
Episodically		180 (11.7%)	160 (12.7%)	20 (7.3%)		0.42 (0.24, 0.72)	**0.002**		
Often		44 (2.9%)	39 (3.1%)	5 (1.8%)		0.43 (0.14, 1.05)	0.091		

^1^ Median (IQR), ^2^ Wilcoxon rank sum test; Pearson’s Chi-squared test, ^3^ OR = Odds Ratio, aOR) Adjusted Odds Ratio, CI = Confidence Interval, * Ancillary workers: cleaning and laundry workers, kitchen staff, drivers, security officer; Administrative workers: secretariat, information technology, accounting, etc.; Other health professionals: radiology, laboratory, pharmacy, etc. ** cancer, chronic heart disease, high blood pressure/hypertension, chronic kidney disease, chronic liver disease, chronic lung disease, diabetes, immunocompromised, neurological disease, obesity. *** Collect a respiratory specimen or sputum specimen, administer a nebulizer, apply nasal cannula, oxygen face mask, or mechanical ventilation, perform tracheal intubation, manual ventilation, suction of fluids or secretions, chest physiotherapy, or bedside bronchoscopy.

**Table 2:** Factors associated with early COVID-19 uptake among nurses and midwives in the multivariable analysis, Georgia, March–July 2021.

		Vaccination Status				Crude Odds Ratio (OR) N = 617		Adjusted Odds Ratio (aOR) N = 617	
Characteristic	Missing n (%)	Total Nurses/Midwives (%) N = 617	Number Unvaccinated (%) N = 540	Number Vaccinated (%) N = 77	*p*-Value ^¹^	OR (95% CI) ^2^	*p*-Value	aOR (95% CI) ^2^	*p*-Value
Hospital study site	0 (0%)				**<0.001**				
Acad. K Central University Hosp.		142.0 (23.0%)	127.0 (23.5%)	15.0 (19.5%)		-		-	
Batumi Republican Hospital		113.0 (18.3%)	78.0 (14.4%)	35.0 (45.5%)		3.80 (1.98, 7.59)	**<0.001**	2.31 (1.16, 4.79)	**0.020**
Bochorishvili Clinic		74.0 (12.0%)	71.0 (13.1%)	3.0 (3.9%)		0.36 (0.08, 1.13)	0.11	0.21 (0.04, 0.76)	**0.030**
Bokeria Tbilisi Referral Hospital		122.0 (19.8%)	112.0 (20.7%)	10.0 (13.0%)		0.76 (0.32, 1.73)	0.5	0.53 (0.21, 1.30)	0.2
Caucasus Medical Centre		120.0 (19.4%)	111.0 (20.6%)	9.0 (11.7%)		0.69 (0.28, 1.60)	0.4	0.49 (0.19, 1.20)	0.13
Infectious Disease Hospital		46.0 (7.5%)	41.0 (7.6%)	5.0 (6.5%)		1.03 (0.32, 2.85)	>0.9	0.52 (0.13, 1.71)	0.3
Vaccinated against influenza	0 (0%)				**<0.001**				
No		429.0 (69.5%)	395.0 (73.1%)	34.0 (44.2%)		-		-	
Yes		188.0 (30.5%)	145.0 (26.9%)	43.0 (55.8%)		3.45 (2.12, 5.64)	**<0.001**	2.38 (1.40, 4.07)	**0.001**
Regular contact with infants at work	0 (0%)				0.3				
No		557.0 (90.3%)	490.0 (90.7%)	67.0 (87.0%)		-		-	
Yes		60.0 (9.7%)	50.0 (9.3%)	10.0 (13.0%)		1.46 (0.67, 2.91)	0.3	3.56 (1.37, 9.01)	**0.008**
COVID-19 vaccination is effective	0 (0%)				**<0.001**				
Not effective		82.0 (13.3%)	80.0 (14.8%)	2.0 (2.6%)		-		-	
Somewhat effective		369.0 (59.8%)	327.0 (60.6%)	42.0 (54.5%)		5.14 (1.54, 31.90)	**0.026**	3.79 (1.07, 24.10)	0.078
Very effective		166.0 (26.9%)	133.0 (24.6%)	33.0 (42.9%)		9.92 (2.91, 62.20)	**0.002**	6.78 (1.84, 44.20)	**0.013**

^¹^ Pearson’s Chi-squared test; Wilcoxon rank sum test; Fisher’s exact test, ^2^ OR = Odds Ratio, aOR = adjusted Odds Ratio CI = Confidence Interval.

## 4. Discussion

In this analysis of enrolment data collected from HWs participating in a COVID-19 vaccine effectiveness cohort study at six large hospitals in Georgia, only 17.9% HWs were vaccinated against COVID-19 with one dose at the time of enrolment, during March–July 2021, a period that overlapped with the first months of the national vaccination campaign. To our knowledge, this is the first study to examine factors associated with early uptake of COVID-19 vaccine in HWs in Georgia.

We found that non-physicians and HWs who had been previously infected with SARS-CoV-2 were significantly less likely to have received the COVID-19 vaccine, whereas older HWs, those who had received seasonal influenza vaccine (winter 2020/2021), and HWs who considered the COVID-19 vaccine highly effective were more likely to have been vaccinated.

Our findings are consistent with previous studies that have reported lower vaccination coverage among non-physicians [6,7,8,9,10,11]. Because we found that nurses and midwives, a large and important category of HWs, had particularly low vaccine uptake compared to physicians, we investigated factors associated with vaccine uptake among nurses and midwives only. We found that some of the same factors associated with increased uptake in the overall cohort, such as confidence in the vaccine’s effectiveness, and previous influenza vaccine, were strong positive predictors of COVID-19 vaccine uptake in this subgroup. In contrast, age and previous SARS-CoV-2 infection were not significantly associated with receipt of COVID-19 vaccine in this occupational group. These results suggest that factors associated with vaccine uptake may slightly differ among HW occupations, and further underscore the importance of tailored public health messaging.

We found that concerns about vaccine safety and vaccine effectiveness, and insufficient knowledge about COVID-19 vaccines were associated with lower COVID-19 vaccine uptake in our study population, findings that have been reported among HWs elsewhere in the world [3]. Our findings also suggest the importance of the perceived benefits of the vaccine, as HWs who thought COVID-19 vaccines were more effective were more likely to be vaccinated. Although the self-reported knowledge and attitudes toward COVID-19 vaccine safety were not associated with vaccine uptake in our analysis, we found widespread concern about vaccine safety, as half of the participants “mostly disagreed” or pronounced themselves as “neutral” with the statement that COVID-19 vaccines are safe. This finding underscores the need to improve messaging about vaccine safety to HWs in Georgia, not just to increase uptake among the HWs themselves, but also to increase the chances that HWs, who are a highly trusted source for vaccine information among the public, share accurate, supportive information about vaccine safety with their patients.

Data from a public opinion survey, conducted in Georgia by NGOs between late April 2020 and February 2021 suggested that intention to get vaccinated in the general population was low and highlighted a general lack of trust in the quality of the COVID-19 vaccine [22,23]. Furthermore, although vaccine preference was not considered in this study, it is plausible that some participants were delaying vaccination until vaccines other than AstraZeneca became available; safety concerns led to the temporary suspension of this vaccine in some European countries, and AstraZeneca was one of the main vaccines available to HWs and the general population in Georgia at the beginning of the vaccination campaign.

Finally, our study corroborates other known factors associated with COVID-19 vaccine uptake in HWs in other settings, such as older age and seasonal influenza vaccination, and those associated with a decrease in COVID-19 vaccine receipt, such as a prior SARS-CoV-2 infection [3,6,7,8,9,10,11]. Although older HWs are at higher risk of severe illness from COVID-19, younger HWs are still at risk of infection and, to a lesser extent, severe disease. However, infections can lead to a depleted workforce in hospitals and clinics, which has been observed widely during the COVID-19 pandemic [24]. Additionally, infected HWs risk transmitting the infection to their vulnerable patients. Public health messaging targeting younger HWs with emphasis on the benefits of vaccination is crucial to increasing vaccine uptake in this group.

Receipt of seasonal influenza vaccine in the 2020–2021 influenza season was found to be a positive predictor of COVID-19 vaccine uptake in our study. This finding suggests that investment and promotion of annual influenza vaccination among HWs might positively affect COVID-19 vaccine acceptance.

We did not find any significant difference in vaccine uptake by sex, occupational exposure, such as care provision or regular contact with vulnerable patient groups at work, or other behavioral factors with respect to public health and social health measures, such as the use of facemasks, physical distancing, and social interactions.

Our study has a number of strengths. We enrolled a large number of HWs—over 1500—from six hospitals in Georgia, and enrolment data were complete for nearly all questions. In addition, we were able to validate all self-reported vaccination data using a comprehensive national vaccine registry.

This study has some limitations. First, the study may suffer from selection bias; while all eligible HWs were invited to participate in the vaccine effectiveness study, the study was voluntary, and participants who chose to participate in the study may not be representative of all HWs at their institutions. In addition, our analysis only included HWs working in hospitals in Tbilisi and Batumi, which may not be representative of HWs in the rest of the country. However, by the end of June 2021, uptake among HWs across Georgia was approximately 19% [25], which is consistent with the uptake at enrolment in our study. Behavioral factors consisted of self-reported variables that focused on the last seven days before the interview and were subject to both recall and social desirability bias. Furthermore, this cross-sectional study offers a snapshot of the early uptake of the COVID-19 vaccine in the first three months of the vaccine rollout. Differences in vaccine uptake over time might have lessened as acceptance changed and more HWs decided to get vaccinated.

## 5. Conclusions

We observed low COVID-19 vaccine uptake among HWs in the first few months that the COVID-19 vaccine was available in Georgia. Older HWs, those previously vaccinated against seasonal influenza, and HWs who considered COVID-19 vaccines highly effective were more likely to have been vaccinated early, whereas HWs who were not physicians, and HWs who had been previously infected with SARS-CoV-2 were significantly less likely to have received the vaccine. Community engagement and a tailored communication campaign addressing non-physicians and younger HWs are critical to increasing vaccine uptake among HWs in Georgia, particularly in light of the continued relatively low rates of COVID-19 vaccine coverage well over a year after the vaccine was first offered in the country. In addition, public health messaging emphasizing the safety and the individual and collective benefits of vaccination could help increase vaccine coverage in a timely manner.

## Data Availability

Restrictions apply to the availability of these data. Data are available from the authors with the permission of the World Health Organization Regional Office for Europe and the National Center for Disease Control and Public Health of Georgia (NCDC).
